# Occurrence, Bioaccumulation, and Risk Assessment of Organophosphate Esters in Rivers Receiving Different Effluents

**DOI:** 10.3390/toxics12080612

**Published:** 2024-08-20

**Authors:** Shuyan Da, Jun Wang

**Affiliations:** 1 Medical College, Yangzhou University, Yangzhou 225009, China; shuyanda@hotmail.com; 2 College of Animal Science and Technology, Yangzhou University, Yangzhou 225009, China

**Keywords:** organophosphate esters, effluent-receiving rivers, occurrences, bioaccumulation

## Abstract

Organophosphate esters (OPEs), as alternatives to brominated flame retardants, are extensively used in both production and daily life, with their environmental contamination and toxic effects being a concern. This study investigated the concentration levels, bioaccumulation, and ecological effects of OPEs in five different effluent-receiving rivers. The results demonstrate that the concentration range of Σ_13_OPEs across the five rivers was between 142.23 and 304.56 ng/L (mean: 193.50 ng/L). The highest pollution levels of OPEs were found in rivers receiving airport and industrial wastewater, followed by agricultural wastewater, mixed wastewater, and domestic wastewater. Tris(2-chloroisopropyl) phosphate (TCPP), triethyl phosphate (TEP), and tricresyl phosphate (TCrP) were identified as the main pollutants. The accumulation concentrations of OPEs in fish ranged from 54.0 to 1080.88 ng/g dw, with the highest bioaccumulation found in *Pelteobagrus fulvidraco*, followed by *Carassius auratus* and *Misgurnus anguillicaudatus*. The brain was the primary organ of accumulation, followed by the liver, gills, intestine, and muscle. Tri-n-propyl phosphate (TPeP) and TEP exhibited the highest bioconcentration, with log BAF values exceeding three. The bioaccumulation of OPEs was influenced by pollutant concentration levels, hydrophobic properties, and biological metabolism. Ecological risk assessment revealed that the cumulative risk values of Σ_13_OPEs ranged from 0.025 to 16.76, with TCrP being the major contributor. It posed a medium–low risk to algae but a high risk to crustaceans and fish.

## 1. Introduction

Organophosphate esters (OPEs) are a class of synthetic industrial chemicals known for their excellent flame retardant and plasticizing properties, comprising halogenated phosphate esters, alkyl phosphate esters, and aromatic phosphate esters. Halogenated phosphate esters are used as flame retardants in products such as furniture, plastics, electronic devices, building materials, and textiles, while alkyl and aromatic phosphate esters are widely applied as plasticizers and antioxidants in plastics, paints, hydraulic fluids, and floor polishes, among others. As ideal substitutes for brominated flame retardants, the usage of OPEs has been increasing annually. In the European Union, usage grew from 85,000 tons in 2005 to 680,000 tons in 2015; in China, the consumption was about 300,000 tons in 2013, with an annual growth rate of 15% [1]. With the widespread production and use of OPEs, they have been continuously detected in natural water bodies, such as rivers, lakes, and coastal areas, accumulating in aquatic organisms and transferring through the food chain, causing various degrees of stress effects on daphnids, fish, rodents, birds, and humans, including endocrine disruption, developmental toxicity, neurotoxicity, and reproductive toxicity [2,3,4].

The detected concentrations of OPEs in aquatic environments vary from ng/L to several tens of μg/L, with domestic wastewater and industrial wastewater discharge being the primary sources of OPEs in water bodies [5]. In rivers receiving effluent from wastewater treatment plants, the concentrations of 10 OPEs ranged from 439.61 to 1053.06 ng/L, with an average concentration of 761.77 ng/L, significantly higher than that in rivers unaffected by effluent discharge [6]. Studies have identified chlorinated OPEs as typical pollutants in wastewater discharged by electronics companies, with tris(phenyl phosphate) (TPhP) having the highest concentration in wastewater from paint and coating companies, tris(n-butyl phosphate) (TnBP) being most concentrated in wastewater from textile industries, and tris(2-chloroisopropyl) phosphate (TCPP) and TPhP showing higher concentrations in wastewater from cardboard and carton industries [7]. Furthermore, tris(butoxyethyl) phosphate (TBEP) has been identified as a major OPE pollutant in domestic wastewater in countries such as the USA [8], Austria [9], and Australia [10], while TiBP, TnBP, TCEP, and tris(1,3-dichloro-2-propyl) phosphate (TDCPP) have been detected at concentrations of up to 1068 ng/L in water bodies receiving agricultural wastewater [11]. The concentration levels and compositional profiles of OPEs in different effluent-receiving water bodies show significant variations, and conducting systematic studies can aid in better understanding the sources and environmental behavior of OPEs.

OPEs are continually ingested by aquatic organisms and accumulate within their bodies, leading to residual levels in fish ranging from several tens to thousands of ng/g lw (lipid weight) [12]. Investigations revealed that in 27 fish species samples from European rivers such as the Adige River, Evrotas River, and Sava River, the total concentration of 14 OPEs ranged between 14.4 and 650 ng/g lw [13], with benthic fish species exhibiting higher accumulation concentrations than pelagic species. In terms of bioaccumulation potential in fish tissues, OPEs were found to accumulate more in liver tissue, followed by kidneys, muscles, gills, etc. [14], demonstrating a certain potential for biomagnification [15]. The characteristics of OPE pollution, bioaccumulation, and ecological risks in water bodies receiving different types of wastewater still require further investigation.

This study selected five different effluent-receiving rivers as research areas, including rivers receiving domestic wastewater, mixed wastewater, agricultural wastewater, airport runoff, and industrial wastewater, to detect the pollution levels of 13 OPEs in water. It analyzed the distribution characteristics and accumulation levels of the OPEs in fish tissues in highly polluted rivers and assessed the ecological risks of OPEs.

## 2. Materials and Methods

### 2.1. Chemicals and Materials

OPE standards and internal standards were all acquired from Wellington Laboratories (Shanghai, China), with purities exceeding 98% (Table S1). Chromatographic-grade methyl alcohol, acetonitrile, and ethyl acetate were purchased from Merck & Co., Inc., Shanghai, China. Glass-fiber filters (D28) and diatomaceous earth were obtained from Thermo Fisher Scientific (San Jose, CA, USA). HLB solid-phase extraction columns (6 cc, 200 mg) were sourced from ANPEL Laboratory Technologies (Shanghai) Co., Ltd., Shanghai, China.

### 2.2. Sample Collection

This study designated five sampling sites in five rivers receiving different effluents, with the specific details provided in Table 1. The water sample collection method adhered to the “Technical Guidance for Water Quality Sampling” (HJ 494-2009) standard [16]. At each sampling point, triplicate water samples (2 L) were collected using clean brown glass bottles in November 2022. The weather was clear during the sampling period. In the highly polluted river S2, three species of fish, including *Carassius auratus*, *Pelteobagrus fulvidraco*, and *Misgurnus anguillicaudatus*, were collected using trawling and cage traps. Both biological and water samples were preserved in foam boxes with dry ice immediately after collection and rapidly transported to the laboratory. Some basic information about the fish is shown in Table S2. The fish were euthanized with a lethal dose of MS-222 neutralized using NaOH. The fish samples were dissected to obtain tissues such as the intestine, liver, brain, and muscle, which were stored in a −80 °C ultra-low-temperature freezer. All treatments involving animals were carried out under the strict guidelines of the Animal Experiment Ethics Committee of Yangzhou University. The water samples were processed within 48 h before testing.

### 2.3. Sample Pretreatment

#### 2.3.1. Water Sample Pretreatment

Approximately 500 mL water samples was filtered through a 0.45 μm glass-fiber filter and then passed through an Oasis HLB column (Waters Corporation, Shanghai, China) at a flow rate of 4 mL/min. Prior to the sample loading, the column was sequentially preconditioned with 5 mL of ethyl acetate, 5 mL of methanol, and 5 mL of ultrapure water. After sample loading, the column was rinsed with 5 mL of deionized water, the rinse was discarded, and the column was dried under vacuum. Subsequently, it was eluted twice with 5 mL of ethyl acetate, maintaining an elution flow rate of 1 mL/min. The collected eluate was evaporated to dryness under rotary evaporation, reconstituted to 1 mL with methanol, and subjected to LC-MS/MS analysis.

#### 2.3.2. Biological Sample Pretreatment

Approximately 1 g dry-weight samples were placed in 10 mL centrifuge tubes and extracted with 10 mL of an acetonitrile–water solution (with a volume ratio of 1:3) using ultrasonic extraction for 30 min. Each mixture was centrifuged at 3000 rpm for 10 min, and the supernatant was collected, filtered through a micro-pore filter, and transferred to a 200 mL beaker. This step was repeated, and the combined extracts were diluted to 200 mL with ultrapure water. After filtration through a 0.45 μm glass-fiber filter, the solution was passed through an Oasis HLB column at a flow rate of 4 mL/min for enrichment, following the same procedure as that for the water samples. The collected eluate was evaporated to dryness under rotary evaporation, reconstituted to 1 mL with methanol, and prepared for LC-MS/MS analysis.

### 2.4. Instrumental Analysis

The liquid chromatography analysis was performed using an ACQUITY BEH C18 column (2.1 mm × 100 mm, 1.7 μm, Waters Corporation, Shanghai, China). The injection volume was set at 5 µL, with a column temperature of 40 °C. The mobile phase consisted of a 0.2% formic acid aqueous solution (A) and methanol (B). A gradient elution method, as detailed in Table S3, was employed for the detection. Mass spectrometry analysis was conducted using a Waters Acquity Xevo TQ Triple Quadrupole Mass Spectrometer under the following conditions: a capillary voltage of 3.0 kV, a collision gas flow rate of 0.16 L/min, and an ion source temperature of 150 °C; a cone gas flow rate of 50 L/h; a desolvation gas temperature of 500 °C; and a desolvation gas flow rate of 900 L/h. Quantitative analysis of the target pollutants was performed using a positive-ion-mode electrospray ionization (ESI+) source and multiple-reaction monitoring (MRM), with the precursor ions, product ions, and collision energies for the target pollutants specified in Table S4.

### 2.5. Quality Control and Assurance

The entire process of the sample collection, sample pretreatment, and sample analysis adhered to strict quality control procedures, accompanied by quality control experiments. The spiking concentrations for the water samples were set at 10 and 100 ng/L and, for the biological samples, at 10 and 100 ng/g dw. Instrument and method stability was ensured before each sample analysis by employing the standard curves of the standard substances, with the target compounds being qualitatively and quantitatively determined based on the characteristic ions and retention times. The target pollutants were not detected or were below the quantification limit in the blank controls. The concentration range for the target pollutants’ standard curves was 0.1 to 200 µg/L (0.1 µg/L, 1 µg/L, 10 µg/L, 50 µg/L, 100 µg/L, and 200 µg/L), with a linear correlation coefficient (*R*^2^) greater than 0.99. The sensitivity of the method for detecting the target pollutants was assessed via the limits of detection (LODs) and limits of quantification (LOQs), where the LODs were defined as the concentration corresponding to three times the signal-to-noise ratio and the LOQs as ten times the signal-to-noise ratio. The LODs for the 13 OPEs ranged between 0.062 and 2.598 ng/L, and for the LOQs, between 0.208 and 8.660 ng/L. The spiking recovery rate ranged from 60.00% to 118.80%, meeting the analytical requirements. The data on the linear range, LODs, LOQs, and recovery rates are shown in Table 2.

### 2.6. Data Analysis and Processing

Following the EMA and the REACH guidelines [17], environmental risk assessments of the OPEs were conducted based on both chronic and acute toxicity data. Typically, the risk quotient (RQ) for OPEs is calculated by dividing the measured environmental concentration (MEC) by the predicted no-effect concentration (PNEC), as shown in Equation (1):

RQ = MEC/PNEC (1)

As per the REACH guidance document, the PNEC was estimated based on the toxicity data. When only short-term/acute toxicity data (EC_50_ or LC_50_) were available, the PNEC was calculated by dividing the EC_50_ or LC_50_ by an assessment factor (AF = 1000). An RQ < 0.01 indicated no significant ecological risk, 0.01 < RQ < 0.1 indicated a low ecological risk, 0.1 ≤ RQ < 1.0 indicated a moderate risk, and RQ ≥ 1.0 indicated a high risk.

This study determined the estimated daily intake (EDI) of OPEs in the captured three fish species to assess the health risk quotient (HQ) for individual OPE exposure in the fish using Equations (2) and (3) [18].

(2) $$\mathrm{EDI}=\sum_{i=1}^{n}C_{\mathrm{orqi}}\times {\mathrm{DC}}_{i}/\mathrm{BW}$$

RQ = MEC/PNEC(3)

where C_orgi_ (ng/g dw) is the concentration of the OPEs in the biological samples, and DC (g/day) is the daily intake of the aquatic organisms, referencing dietary surveys conducted in China [19]. BW denotes body weight, with adult men and women (>18 years) assumed to weigh 65.6 kg and 56.5 kg, respectively; adolescent males and females (6–18 years) assumed to weigh 41.7 kg and 39.0 kg, respectively; and male and female children (2–5 years) assumed to weigh 16.8 kg and 16.0 kg, respectively. The Rfd (reference dose) was referenced from other studies [20]. According to the standards set by the United States Environmental Protection Agency, an HQ > 1 indicated a high health risk from OPEs; an HQ between 0.1 and 1 indicated a moderate risk; an HQ between 0.01 and 0.1 indicated a low risk; and an HQ < 0.01 indicated no health risk [21].

Furthermore, the hazard index (HI) for the OPEs was calculated by summing the HQ for each OPE as per the following formula [18]:

HI = ΣHQ (4)

The bioaccumulation factor (BAF) was used to predict the bioaccumulation potential of the chemical pollutants in aquatic organisms and the resulting potential toxicity [22]. The BAF (L/kg) was determined by the ratio of the concentration in the biota to the concentration in the water, with the following calculation formula:

BAF = C_biota_/C_water_ (5)

If the bioaccumulation coefficient in aquatic organisms exceeded 5000, i.e., with a log BAF of greater than 3.7, the chemical was defined as “bioaccumulative”; if 3.3 < log BAF < 3.7, it was defined as having “potential bioaccumulative” properties [23].

## 3. Results and Discussion

### 3.1. OPE Contamination in Aquatic Environments

**Figure 1:**
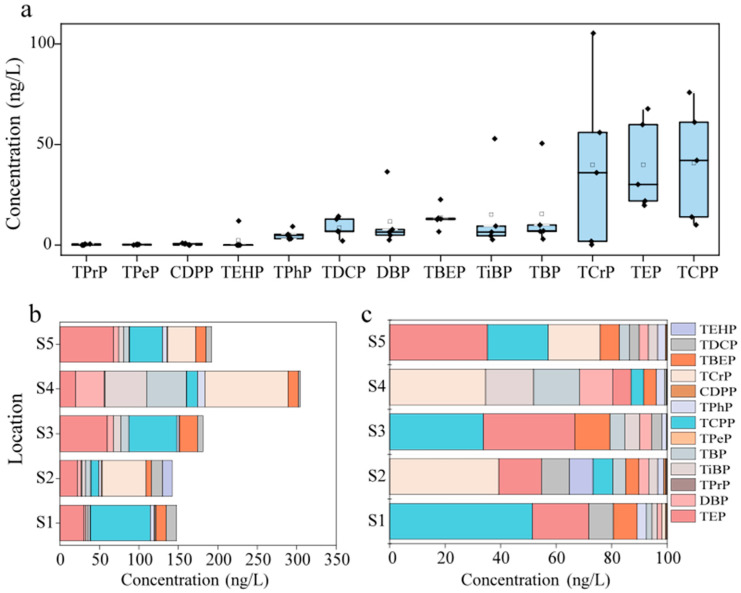
Concentrations of OPEs in the water samples (ng/L) at each sampling site (**a**); composition of OPEs in different water bodies (**b**) and contribution rate (**c**).

In five effluent-receiving rivers, all 13 OPEs were detected with individual concentrations ranging from 0.22 ng/L to 105.33 ng/L and an average concentration of 14.88 ng/L. The overall detected levels in water are shown in Table S5. As illustrated in Figure 1a, the nine OPEs with higher measured concentrations, in descending order, were TCPP, TEP, TCrP, TBP, TiBP, TBEP, DBP, TDCP, and TPhP, all with a detection rate of 100%, followed by TPeP (87.5%), CDPP (37.5%), TPrP (25%), and TEHP (12.5%).

The distribution of the OPEs in the different effluent-receiving water bodies varied (Figure 1b,c), with TCPP, TEP, and TCrP showing the highest average contribution rates of 23.75%, 22.15%, and 18.81%, respectively, across the five types of effluent-receiving water bodies. In rivers receiving industrial wastewater, the contribution rate of TEP reached 35.28%, followed by TCPP (21.91%) and TCrP (18.74%). In rivers receiving domestic wastewater, TCPP accounted for 51.43%, with TEP and TDCP contributing 20.44% and 8.73%, respectively. In rivers receiving agricultural wastewater, TCPP and TEP each accounted for approximately 33%, significantly higher than TCrP (0.12%), whereas TCrP was mainly detected in airport runoff- and mixed wastewater-receiving rivers, with contribution rates of 39.4% and 34.6%, respectively. TCPP and TEP, as typical halogenated alkyl and alkyl OPEs, are widely used as flame retardants in plastic products, polyurethane foam, and textiles and are frequently utilized in industrial, agricultural, and everyday life [24]. In a wastewater treatment plant in Beijing, the cumulative concentration of TCPP and TEP accounted for 24.22% of the total concentration, with TCPP exceeding TEP [25], whereas in an effluent-receiving river in Chengdu, China, the contribution rate of TCPP was only 12%, but TBEP and TCPP contributed up to 63%. Additionally, in the airport runoff-receiving water bodies in this study, TiBP (17.37%) and TBP (16.60%) had a larger share, indicating a higher specificity, and differed from the components of OPEs in a river near an airport in New York, USA, where TCIPP reached concentrations of 427 ng/L [26].

Considering the total concentration of the OPEs in the receiving rivers, the highest total concentrations of the OPEs were found in rivers receiving airport runoff and industrial wastewater, at 304.56 ng/L and 192.08 ng/L, respectively, followed by agricultural wastewater (181.06 ng/L), domestic wastewater (147.56 ng/L), and mixed wastewater (142.23 ng/L). The concentrations of pollutants in rivers receiving airport runoff and industrial wastewater were lower than those reported in previous studies for factory effluent (3000–18,000 ng/L, with an average concentration of 6634.17 ng/L) and airport runoff (174~24,600 ng/L, with an average of 1250 ng/L) [5]. Compared with human habitation areas, dense industrial production inputs more OPEs, causing point-source pollution of OPEs [27]. In addition to typical industrial areas, the environment around airports contains more OPEs, as the widespread use of OPEs in aircraft lubricants and hydraulic oils leads to aircraft emissions being a significant source of OPEs around airports.

### 3.2. Accumulation Characteristics of OPEs in Typical Fish from Urban Water Bodies

#### 3.2.1. Concentration Distribution of OPEs in Fish

A total of 12 OPEs were detected in fish, with individual concentrations ranging from 0.28 to 617.25 ng/g dw and an average concentration of 41.61 ng/g dw. Only TEP and TCPP reached a detection rate of 100%, while the detection range for TPrP, TBP, TPeP, TDCP, TiBP, TPHP, TCrP, CDPP, DBP, and TEHP was between 22.0% and 94.0%, with TBEP not detected. In terms of its bioaccumulation potential, TEP exhibited the highest concentration in *Pelteobagrus fulvidraco*, reaching 617.25 ng/g dw, followed by *Carassius auratus* (346.46 ng/g dw) and *Misgurnus anguillicaudatus* (37.26 ng/g dw), contributing to 61.96% in fish. It is suggested that the high pollution concentration of TEP in water and its wide range of intake pathways in fish, along with a slower rate of in-body degradation and metabolism, are the main reasons for its high accumulation [28]. Detailed information is provided in Table S6.

The highest total concentration of OPEs was found in *Pelteobagrus fulvidraco*, reaching 1080.88 ng/g dw, followed by *Carassius auratus* (487.82 ng/g dw) and *Misgurnus anguillicaudatus* (54.00 ng/g dw) (0a). As a benthic omnivorous fish, *Pelteobagrus fulvidraco* accumulated 11 kinds of OPEs, showing high bioaccumulation potential for TEP, TPhP, and TDCP, with contribution rates of 57.11%, 9.73%, and 8.10%, respectively. The concentration of OPEs in *Pelteobagrus fulvidraco* was 2.2 and 20 times higher than that in *Carassius auratus* and *Misgurnus anguillicaudatus*, respectively, aligning with previous findings that benthic organisms accumulate higher concentrations of OPEs than pelagic aquatic organisms [29]. *Carassius auratus* contained 11 detected monomers, with the highest concentrations found for TEP (346.46 ng/g dw), TPeP (57.46 ng/g dw), and TCPP (30.83 ng/g dw), contributing 71.02%, 11.78%, and 6.32%, respectively. *Misgurnus anguillicaudatus*, being a benthic organism, showed the lowest bioaccumulation of OPEs, primarily comprising TEP (37.26 ng/g dw), TPrP (5.60 ng/g dw), and TCPP (4.98 ng/g dw), with contribution rates of 69.00%, 10.37%, and 9.22%, respectively. Studies indicate that chlorinated OPEs like TCPP, TDCP, and high-molecular-weight OPEs like TPhP are the most common in sediments [30], which are found in higher concentrations in *Misgurnus anguillicaudatus* and *Pelteobagrus fulvidraco*. Additionally, TDCP and TPhP, with high hydrophobicity (*K_ow_* values of 3.65 and 4.7, respectively) and large molecular weights (326.29 g/mol and 430.91 g/mol, respectively), tend to accumulate in organisms [31]. The bioaccumulation of OPEs is related to species specificity, living habits, and the bioaccumulation and bioavailability of pollutants.

**Figure 2:**
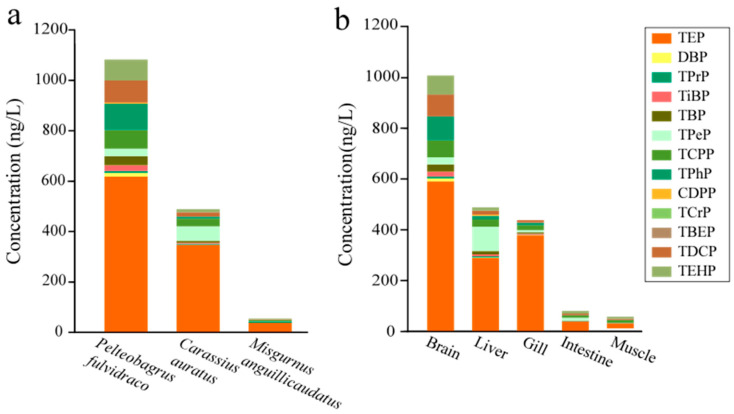
Concentrations of OPEs in typical fish (ng/g dw) (**a**) and in different tissues of fish (ng/g dw) (**b**).

The range of OPE accumulation in various tissues was 45.52–1007.56 ng/g dw, with the highest accumulation observed in the brain (1007.56 ng/g dw), followed by the liver (487.26 ng/g dw), gills (437.34 ng/g dw), intestines (78.85 ng/g dw), and muscles (45.52 ng/g dw) (Figure 2b). The main OPEs in the brain included TEP (589.77 ng/g dw) > TPhP (94.77 ng/g dw) > TDCP (86.43 ng/g dw) > TEHP (75.00 ng/g dw), with TEP contributing 58.53%, and other monomers contributing less than 10%. These substances can cross the blood–brain barrier, leading to neurotoxicity and interference with neural progenitor cell proliferation [32]. The highest concentration of TEP in the liver reached 287.76 ng/g dw, followed by TPeP (95.42 ng/g dw) and TCPP (29.53 ng/g dw), with respective contribution rates of 59.06%, 19.58%, and 6.06%. The liver, as the primary site of metabolism, is where pollutants first accumulate and metabolize via the bloodstream [33]. Previous studies have also demonstrated that the liver has a higher potential for OPE accumulation compared with muscles and gills, primarily influenced by hepatic cytochrome enzymes, blood proteins, and their binding with OPEs [34]. The concentrations of OPEs in the brain and liver are higher than those detected in carp tissues from Lake Taihu (∑_19_ OPEs ranged from 0.833 to 32.3 ng/g dw) [35]. In gills, intestines, and muscle tissues, TEP remained the OPE with the highest contribution rate, accounting for 86.36%, 45.60%, and 41.65%, respectively. The concentration of TEP in the gills reached 377.72 ng/g dw, which was 20 times higher than the second highest, TCPP, and significantly exceeded the TEP concentrations in the intestines (35.96 ng/g dw) and muscles (18.96 ng/g dw). A comparative analysis of the concentration differences in the OPEs between the gills and intestine, two absorptive organs, indicated that gill filtration is an important pathway for fish to accumulate OPEs. The lipid content in tissues and the tissue/blood partition coefficient play significant roles in the distribution of hydrophobic compounds [34], with the lower lipid content in muscles being one of the factors leading to the lower accumulation of OPEs in muscles.

#### 3.2.2. BAFs of OPEs

**Figure 3:**
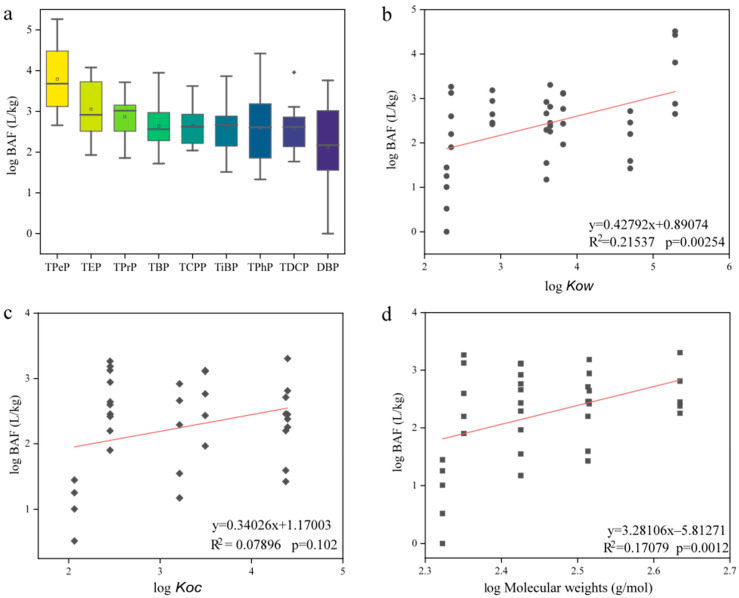
Log BAFs of OPEs (**a**); linear relationships between log BAF and log *Kow* values of OPEs (**b**); linear relationships between log BAF and log *Koc* values of OPEs (**c**); linear relationships between log BAF and log molecular weight values of OPEs (**d**).

The BAFs of the OPEs were calculated based on the concentrations of the OPEs in fish and water (detailed information is provided in Table S7), as shown in Figure 3a. The results show that the average log BAF values for the 13 OPEs in fish tissues ranged from 0.70 to 3.38, with TPeP and TEP having the highest values, at 3.37 and 3.05, respectively, indicating potential bioaccumulative properties. Similar patterns were observed in a bioaccumulation survey of fish in Laizhou Bay, China, where the log BAF range for TPeP was 1.9–4.3 (average: 3.5), while the BAFs for other OPEs ranged between 2.67 and 3.25 [14]. Analysis of the correlation between the log BAF values of OPEs and their log *K_ow_*, log *K_oc_*, and molecular weight (0b–d) revealed a positive correlation between the bioaccumulation potential of the OPEs and their hydrophobicity and molecular weight, whereas the correlation with adsorption capacity was not significant. Previous studies found that TPeP (log *K_ow_* = 5.29), despite lower detection concentrations in water, exhibited the highest BAF, whereas TCPP (log *K_ow_* = 2.89), with higher detection concentrations and lower lipophilicity, showed lower bioaccumulation [36]. The molecular weight of compounds, to some extent, affects the accumulation and distribution of OPEs in organisms; hydrophobicity increases with molecular weight, leading to a lower water solubility and higher bioaccumulation potential for compounds with larger molecular weights [28]. Additionally, the log BAF values for the other eight OPEs were all less than 3.3, indicating no bioaccumulative properties. The bioaccumulative potential of OPEs is related to their low concentrations as well as the diet and metabolism of organisms. Lower exposure concentrations of OPEs can enhance bioaccumulation within organisms, while higher exposure levels may promote metabolic processes and reduce bioaccumulation [37].

### 3.3. Risk Assessment of OPEs

#### 3.3.1. Ecological Risk Assessment Based on RQ

**Figure 4:**
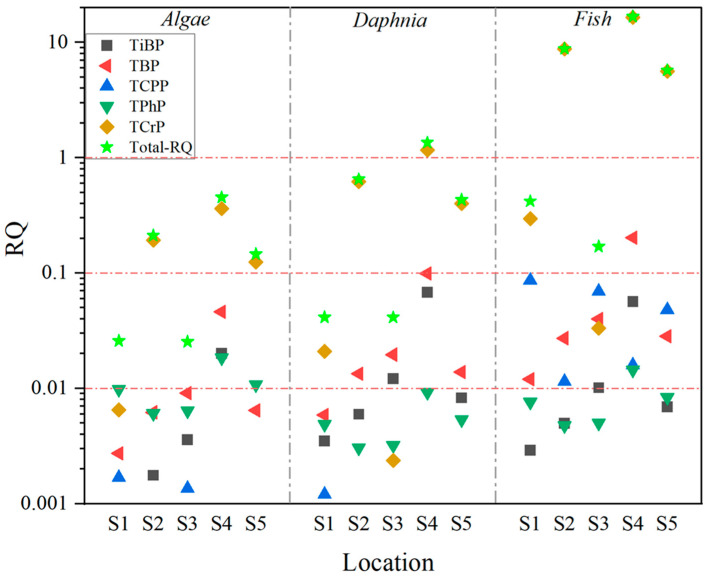
Ecological risk quotient of OPEs.

The ΣRQ range of the OPEs in the different effluent-receiving rivers was 0.025–16.76 (0). In rivers receiving agricultural and domestic wastewater, the ΣRQ values were less than 1, posing a medium ecological risk to aquatic life, while mixed wastewater-, airport runoff-, and industrial wastewater-receiving rivers had ΣRQ ranges of 0.21–8.80, 0.45–16.75, and 0.15–5.73, respectively, all indicating a high ecological risk. For the aquatic organism types, the mixed risk ranges of the OPEs for algae, daphnia, and fish were 0.025–0.45, 0.04–1.35, and 0.17–16.76, respectively, increasing with the trophic level (Figure 4). TCrP was the primary risk compound for algae, daphnia, and fish, with contribution rates of 79.80%, 88.00%, and 97.72%, respectively; the risk contribution of TCrP increased with the trophic level of the aquatic organisms [20]. Fish are the most sensitive species to OPE exposure, and prolonged exposure in effluent-receiving rivers could lead to negative effects, such as delayed hatching, cardiac edema, and craniofacial abnormalities [38]. Among the 13 OPEs detected in water bodies, TCrP posed a medium risk to algae and daphnia, with average risk values of 0.14 and 0.44, respectively, and a high risk to fish (with an average RQ of 6.23). Four compounds, TBP, TCPP, TiBP, and TPhP, posed a low–medium risk to aquatic life, while the other OPEs showed no significant ecological risk. The ecological risk of TCrP to algae and daphnia mainly manifests as growth and reproduction inhibition, which may reduce fish behavioral performance under short-term exposure [6], whereas TBP inhibits growth, reproduction, locomotor behavior, and metabolic enzyme activity in fish [39].

#### 3.3.2. Human Health Risk Assessment

**Figure 5:**
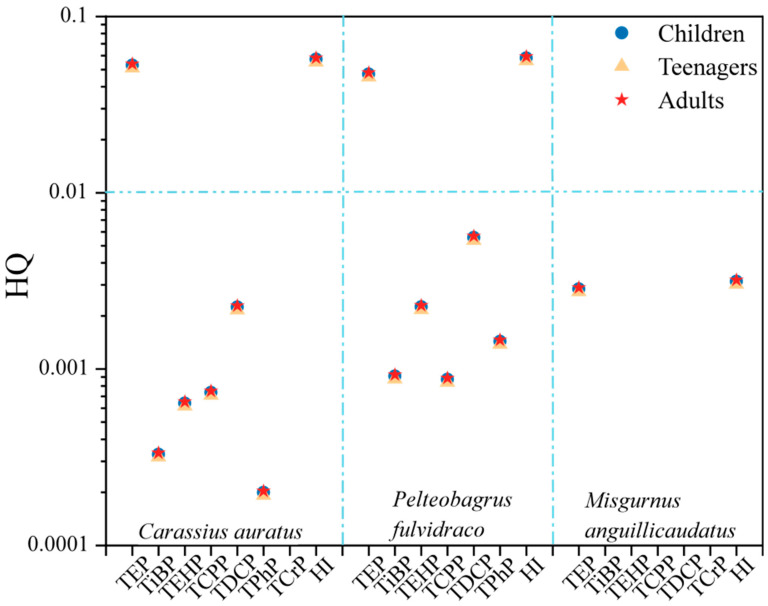
Health risk quotient (HQ) and hazard index (HI) for exposure of three fish species to different populations.

Based on the available Rfd values for seven OPEs, the HQ and total HI for different age and gender groups consuming three types of fish were calculated, as shown in Figure 5. The average non-carcinogenic health risk coefficients (HQ) from consuming the three types of fish were ranked as follows: *Pelteobagrus fulvidraco* (8.29 × 10^−3^) > *Carassius auratus* (8.14 × 10^−3^) > *Misgurnus anguillicaudatus* (4.50 × 10^−4^), with adults having slightly higher HQ values than adolescents and children, ranging between 2.4 × 10^−5^ and 5.4 × 10^−2^, with no significant differences observed between genders within the same age group.

TEP was the main contributor to non-carcinogenic risk, contributing between 81.02% and 92.63%, leading to a low level of non-carcinogenic health risk (HQ > 0.01) after consumption of *Carassius auratus* and *Pelteobagrus fulvidraco*. The combined health risk values (HI) for OPEs ranged from 0.0031 to 0.058, below the safety threshold of 1, indicating no severe health harm [7]. This study assessed the health risks for only seven OPEs, but with the diversity and increasing use of OPEs in the environment, human residual levels are also rising. This not only damages adolescent thyroid function [40] and affects fetal gestational age and weight [41] but also poses reproductive developmental effects, transgenerational toxicity, and other health impacts on humans [42]. Further attention is needed on the continuous accumulation of OPEs in the environment and organisms, evaluating their health risk pathways to reduce harm to human health.

## 4. Conclusions

This study investigated the occurrence of OPEs in water and fish samples from rivers receiving different effluents, exploring their distribution, bioaccumulation, and ecological and health risks. It was found that airport runoff and industrial wastewater were the main sources of OPEs in the environment, presenting higher concentrations compared with agricultural and domestic wastewater. TCPP, TEP, and TCrP were the predominant OPE pollutants across the effluent-receiving waters, with similar contribution rates among different types of effluents. TEP was the primary pollutant bioaccumulated in organisms, with benthic fish species *Pelteobagrus fulvidraco* showing higher OPE contents than *Carassius auratus* and *Misgurnus anguillicaudatus*, while brain tissue exhibited the highest bioaccumulation potential for OPEs, followed by liver > gills > intestine > muscle. TEP and TPeP demonstrated the strongest bioaccumulative properties, with other pollutant monomers showing nearly no bioaccumulation. The ecological risk assessment indicated that OPEs posed medium–high ecological risks to aquatic life, with risk levels increasing with trophic levels. TCrP was identified as the main contributor to ecological risk, presenting significant toxicological risks to higher aquatic organisms. However, the HQ values suggested that the OPEs currently do not pose a health risk to humans.

## Figures and Tables

**Table 1:** The information about sampling sites.

Sampling Site	Location	River Type
S1	118.85886° E, 30.00257° N	Domestic wastewater
S2	118.73142° E, 32.07486° N	Municipal effluent
S3	118.89894° E, 31.85563° N	Farmland backwater
S4	118.84627° E, 31.72774° N	Airport wastewater
S5	118.53055° E, 31.96582° N	Industrial effluents

**Table 2:** LOQs and LODs of 13 OPEs and their recovery rates in water and organisms.

Compound	Abbr.	LODs	LOQs	Water Samples (ng/L)		Biological Samples (ng/g)	
		ng/L		10.0	100.0	10.0	100.0
Triethyl phosphate	TEP	0.062	0.208	84.8	80.0	90.4	111.52
Phosphoric acid, dibutyl ester	DBP	0.23	0.765	95.2	84.0	96.8	75.6
Tripropyl phosphate	TPrP	1.875	6.250	95.2	80.0	94.4	103.07
Triisobutyl phosphate	TiBP	1.728	5.760	90.4	75.2	95.6	80.53
Tributyl phosphate	TBP	2.346	7.820	115.2	84.8	95.2	118.8
Tripentyl phosphate	TPeP	0.192	0.640	109.6	79.2	102.8	79.87
Tris(2-chloroisopropyl)phosphate	TCPP	0.507	1.690	82.4	60.0	82.0	67.73
Triphenyl phosphate	TPhP	2.598	8.660	104.0	77.6	99.6	96.8
Cresyl diphenyl phosphate	CDPP	1.824	6.008	90.0	73.6	79.4	76.13
Tris(methylphenyl) phosphate	TCrP	0.711	2.370	87.2	107.04	96.88	84.0
Tris(2-butoxyethyl) phosphate	TBEP	0.999	3.330	92.8	114.4	78.0	90.2
Tris(1,3-dichloropropyl)phosphate	TDCP	1.281	4.270	80.8	92.8	98.0	92.4
Tris(2-ethylhexyl)phosphate	TEHP	1.506	5.020	80.4	83.2	86.4	84.2

## Data Availability

The data presented in this study are available on request from the corresponding author.

## Supplementary Materials

The following supporting information can be downloaded at https://www.mdpi.com/article/10.3390/toxics12080612/s1, Table S1: Physical and chemical properties of thirteen organophosphates; Table S2: Basic information of wild fish from different receiving rivers of Nanjing; Table S3: OPEs liquid chromatography gradient elution setup parameters; Table S4: The main monitoring parameters of LC-ESI+-ms/ms for OPEs; Table S5: Overall detection levels of thirteen OPEs in water bodies; Table S6: Concentration of OPEs in each part of the fish body (ng/g dw); Table S7: Three types of wild fish tissue OPEs bioaccumulation factors (BAFs).
