# Supplementary material for: Occurrence, Bioaccumulation, and Risk Assessment of Organophosphate Esters in Rivers Receiving Different Effluents
Source: Toxics. 2024 Aug 20;12(8):612. doi: 10.3390/toxics12080612 (PMC11359139; doi:10.3390/toxics12080612)
Supplement: Supplementary file 1 [file toxics-12-00612-s001.zip › toxics-3144992-supplementary.pdf]

Supplementary information

contents

Table S1. Physical and chemical properties of thirteen organophosphates.

Table S2. Basic information of wild fish from different receiving rivers of Nanjing.

Table S3. OPEs liquid chromatography gradient elution setup parameters.

Table S4. The main monitoring parameters of LC-ESI+-ms/ms for OPEs.

Table S5. Overall detection levels of thirteen OPEs in water bodies.

Table S6. Concentration of OPEs in each part of the fish body (ng/g dw).

Table S7. Three types of wild fish tissue OPEs bioaccumulation factors (BAFs).

Table S1 Physical and chemical properties of 13 OPEs [8]

Compounds	Abbr	log*k*ow	log*k*oc	Solubility (mg/L)	Boiling point (°C)	Melting point (°C)	Vapour pressure (mm Hg)	HLCS (atm/m3/mol)
Tris(2-ethylhexyl)phosphate	TEHP	9.49	6.87	0.6	220	87	2.0×10^{-6}	9.6×10^{-5}
Triethyl phosphate	TEP	0.8	1.68	5×10^{5}	216	−56	3.9×10^{-1}	3.5×10^{-6}
Triphenyl phosphate	TPhP	-	-	-	245	50	1×10^{-2}	-
Tris(methylphenyl) phosphate	TCrP	5.11	-	-	410	-33	1.33	-
Tris(2-butoxyethyl) phosphate	TBEP	3.75	3.01	1.2×10^{3}	414	−70	2.1×10^{-7}	1.3×10^{-11}
Triisobutyl phosphate	TiBP	3.6	3.05	3.72	264	16	1.3×10^{-2}	2.8×10^{-4}
Tris(1,3-dichloropropyl)phosphate	TDCP	3.85	2.35	1.5	457	88	7.4×10^{-8}	2.6×10^{-9}
Tributyl phosphate	TBP	4	3.28	280	289	−80	1.2×10^{-3}	1.8×10^{-4}
Tris(2-chloroisopropyl)phosphate	TCPP	2.59	2.21	1.6×10^{3}	359	72	1.9×10^{-6}	6.0×10^{-8}
Cresyl diphenyl phosphate	CDPP	4.51	3.95	1.9	235	−38	4.7×10^{-6}	4.4×10^{-8}
Phosphoric acid, dibutyl ester	DBP	2.29	-	-	250	-	4.3×10^{-9}	-
Tripropyl phosphate	TPrP	1.87	2.83	827	254	17	2.9×10^{-2}	8.2×10^{-6}
Tripentyl phosphate	TPeP	5.29	-	2.9	-	-	2.2×10^{-3}	-

Table S2 Basic information of wild fish from different receiving rivers of Nanjing

Fish species	Latin name	Feeding habits	Number	Length (cm)[a]	Weight (g)[a]
Crucian carp	*Carassius auratus*	omnivorous	12	19.2±0.9	19.2±0.9
Banded Catfish	*Pelteobagrus fulvidraco*	omnivorous	11	248.4±24.1	436.7±86.5
Pond loach	*Misgurnus anguillicaudatus*	omnivorous	9	13.5±3.4	224.5±32.3

[a]： The value was displayed as mean ± SD.

Table S3 OPEs liquid chromatography gradient elution setup parameters

Time (min)	Velocity (mL/min)	Mobile phase A (0.2% formic acid aqueous solution)	Mobile phase B (methanol)
0-0.25	0.4	70%	30%
0.25	0.4	70%	30%
3	0.4	5%	95%
4	0.4	5%	95%
5	0.4	70%	30%
6	0.4	70%	30%

Table S4 Main monitoring parameters of LC-ESI+-MS/MS for OPEs

Compounds	Standing time (min)	Precursor ions (m/z)	Product ions (m/z)	Cone-hole voltage (v)	Collision energy (v)
TEP	0.161	182.9	98.79/126.87	22	18/12
DBP	0.015	210.97	98.77/154.86	34	10/6
TPrP	0.036	225.28	98.85/140.95	16	16/10
TiBP	0.015	267.03	98.82/154.90	28	18/10
TBP	0.015	267.32	98.84/154.95	18	18/10
TPeP	0.040	209.44	98.84/239.15	22	20/10
TCPP	0.036	326.90/329.00	99.00	15	30
TPhP	0.015	327.29	76.87/215.05	48	38/28
CDPP	0.015	341.10	229.20/265.10	30	20
TCrP	0.015	368.97	90.81/164.99	48	32/50
TBEP	0.015	399.40	199.22/299.33	30	16/12
TDCP	0.015	428.83/430.83	98.82	28	26/24
TEHP	0.015	435.17	98.78	24	14

Table S5 Overall detected levels of 13 OPEs in water

Compounds	Detection rate	Range（ng/L）	Average（ng/L）	Medial（ng/L）
TEP	100%	19.78-68.67	42.98	35.67
DBP	100%	2.17-36.44	9.20	3.11
TPrP	29%	ND-1.09	0.23	0.00
TiBP	100%	2.22-52.89	11.85	3.22
TBP	100%	2.44-50.56	11.93	3.56
TPeP	86%	ND-0.44	0.25	0.22
TBEP	100%	3.80-22.66	13.02	13.67
TEHP	0%	ND	0.00	0.00
TDCP	100%	2.00-13.00	8.03	7.83
TCPP	100%	10.98-80.67	46.00	61.06
TPhP	100%	3.19-9.22	5.20	4.67
CDPP	43%	ND-1.85	0.66	0.00
TCrP	100%	0.21-108.91	41.46	2.97

Table S6 OPEs concentration in different parts of fish (ng/g dw)

Name	Tissue	TEP	DBP	TPrP	TiBP	TBP	TPeP	TCPP	TPhP	CDPP	TCrP	TBEP	TDCP	TEHP	Sum
Carassius auratus	Liver	80.325	ND	1.695	1.16	3	47.43	10.81	5.66	ND	ND	ND	4.69	12.77	158.315
	Intestine	13.345	ND	0.35	0.71	0.705	1.24	2.39	0.35	ND	ND	ND	1.415	4.06	22.015
	Muscle	5.36	ND	0.26	0.07	0.185	0.295	4.2	0.145	ND	0.37	ND	1.84	3.31	15.805
	Brain	209.465	ND	1.39	2.33	1.855	7.88	10.17	3.1	ND	ND	ND	7.8	ND	241.275
	Gill	37.965	0.445	0.5	1.425	1.675	1.24	3.265	2.63	ND	0.5	ND	1.89	ND	50.405
Pelteobagrus fulvidraco	Liver	127.12	2.26	1.13	5.08	7.34	0.56	7.91	8.47	4.52	ND	ND	6.21	ND	170.62
	Intestine	9.27	ND	0.64	0.32	0.96	8.95	4.47	0.64	ND	ND	ND	3.51	6.07	34.82
	Muscle	8.24	0.08	0.08	0.23	0.54	0.23	1.79	0.16	ND	ND	ND	1.01	1.55	13.91
	Brain	170.83	12.50	4.17	16.67	25.00	12.50	45.83	91.67	ND	ND	ND	70.83	75.00	525.00
	Gill	301.80	ND	1.20	0.60	1.80	7.78	13.17	4.19	ND	ND	ND	5.99	ND	336.53
Misgurnus anguillicaudatus	Brain	6.51	ND	ND	ND	ND	ND	1.20	ND	ND	0.23	ND	0.46	2.74	11.14
	Muscle	22.64	ND	ND	0.28	0.28	0.42	1.33	ND	ND	ND	ND	1.47	ND	26.42
	Internal-organs	8.10	ND	5.60	ND	ND	0.17	2.45	ND	ND	0.12	ND	ND	ND	16.44

Table S7 OPEs Bioaccumulation factors (BAFs) of OPEs in tissues of different fish

Name	Tissue	TEP	DBP	TPrP	TiBP	TBP	TPeP	TCPP	TPhP	CDPP	TDCP
Carassius auratus	Liver	3171.5	0	1557	508.5	1061.5	145444.5	985	813.5	0	599.5
	Intestine	527	0	325.5	155	250.5	3798	217.5	51	0	180.5
	Muscle	211.5	0	237.5	16	65	901	382	42	0	235
	Brain	8271	0	1277.5	509.5	657	24166.5	926.5	445.5	0	997
	Gill	1499	203.5	229	624	592	1907	297.5	755	0	242
Pelteobagrus fulvidraco	Liver	5019	1040	1040	2228	2599	1733	720	2436	2446	794
	Intestine	366	0	588	140	339	27433	407	184	0	449
	Muscle	325	36	71	102	192	715	163	45	0	129
	Brain	6745	5750	3833	7302	8846	38333	4175	26354	0	9051
	Gill	11916	0	1102	262	636	23872	1200	1205	0	765
Misgurnus anguillicaudatus	Brain		0	0	0	0	0	109	0	0	58
	Muscle	894	0	0	122	99	1286	121	0	0	188
	Internal-organs	320	0	5150	0	0	536	223	0	0	0
